# Sensitivity of Lung Resistance and Compliance to Beta-Blocker Induced Bronchoconstriction and Long Acting Beta-Agonist Withdrawal in COPD

**DOI:** 10.1007/s00408-017-0079-1

**Published:** 2017-12-20

**Authors:** Sunny Jabbal, Brian J. Lipworth

**Affiliations:** Scottish Centre for Respiratory Research, Ninewells Hospital and Medical School, Dundee, Scotland DD1 9SY UK

**Keywords:** COPD, Bronchoconstriction, Beta-blocker, IOS, Oscillometry

## Abstract

Little is known about impulse oscillometry (IOS) in COPD. IOS is an effort independent measure of lung resistance and reactance (compliance). We assessed how frequency dependence of resistance (R) and reactance (X) changed in response to bronchoconstriction with carvedilol followed by long acting beta-agonist (LABA) withdrawal. *N* = 12 patients with moderate to severe COPD were analysed, who had ≥ 100 ml fall in FEV_1_ with carvedilol. Compared to baseline taking ICS/LABA there were 21, 59, and 135% significant changes in resistance at 5 Hz (R5), reactance at 5 Hz (X5), and reactance area (AX), respectively, with carvedilol, while after LABA withdrawal only AX showed a further significant increase to 210% (i.e. reduced compliance). Hence changes in lung compliance rather than resistance play a more important role in the beta-2 receptor-mediated responses in COPD.

## Introduction

Little is known about impulse oscillometry (IOS) in COPD. Current diagnosis and management of the disease relies heavily on spirometry, specifically the forced expiratory volume in 1 s (FEV_1_) and forced vital capacity (FVC) to assess severity of airflow obstruction. Spirometry unlike IOS is effort dependent and is therefore susceptible to artificial effects of forced expiratory airway closure. IOS is more physiologic being performed with normal tidal breathing, using sound waves of varying frequencies to detect changes in respiratory resistance (R) and reactance (X). In this regard, reactance and compliance are related in reciprocal manner. Changes in airway compliance are thought to reflect the degree of hyperinflation in COPD [1].

A post hoc analysis of ECLIPSE in 2054 patients with COPD demonstrated that airway resistance at 5 Hz but not 20 Hz increased modestly comparing GOLD groups 2 and 4, but airway compliance (as integrated area of low frequency reactance, AX) increased by a much larger degree [2]. Furthermore healthy controls and smokers had mean AX values of 0.38 and 0.34 kPa/L/s Hz, whereas even GOLD group 2 patients had a mean AX of 1.37 kPa/L/s Hz, in turn suggesting that reduced lung compliance plays an important role in the early pathophysiology of the disease.

Airway compliance as AX or reactance at 5 Hz (X5) has been shown to be closely associated with changes in FEV_1_ [2, 3], more so than resistance as R5. There are few data on changes in IOS in response to bronchoconstrictor stimuli in patients with COPD. In one study, there was discordance between R5 and X5 in response to methacholine bronchoconstriction in COPD, while in asthma there was concordance in response [4].

## Methods

Here, we report on a post hoc responder analysis of the carvedilol arm of NCT01656005 [5]. This study had favourable opinion from the East of Scotland Research and Ethics Committee (12/ES/0054). In brief, patients with moderate to severe COPD received an initial run-in of 2 weeks with ICS/LABA as beclometasone 100 µg/formoterol 6 µg 2 puffs bid (Fostair, Chiesi, Manchester, UK). At baseline after run-in, while still receiving ICS/LABA, patients received weekly dose titration with the non-selective beta-blocker carvedilol to achieve a target dose of 12.5 mg bid, which was then given in conjunction with a further 1 week on ICS/LABA. This was followed by LABA withdrawal with a further week to ICS alone as beclometasone 200 µg 2 puffs bid (Clenil, Chiesi) in the presence of continued carvedilol. Patients had lung function tests at baseline (SuperSpiro, Micro Medical Ltd, Chatham, Kent, United Kingdom) and IOS (Jaeger Masterscreen IOS, Hochberg, Germany) on no beta-blocker while taking ICS//LABA and subsequently while taking carvedilol in conjunction with ICS/LABA and ICS alone.

We carried out a responder analysis, selecting those patients (*n* = 12) who exhibited a decrease in FEV_1_ of ≥ 100 ml, i.e. above the MCID [6] in response to carvedilol while taking concomitant ICS/LABA. We calculated percentage change in AX, X5, R5, R20, FEV_1_ and FVC comparing pre beta-blocker baseline (on ICS/LABA) to carvedilol plus either ICS/LABA or ICS alone. We also calculated standardised response means (SRM), which are a relative measure of effect size and responsiveness and so express the signal (mean)-to-noise (standard deviation) ratio [7]. An SRM of 0.8 units or greater is considered highly sensitive, and because they are standardised scores, they can be compared between variables [7].

## Results

**Fig. 1:**
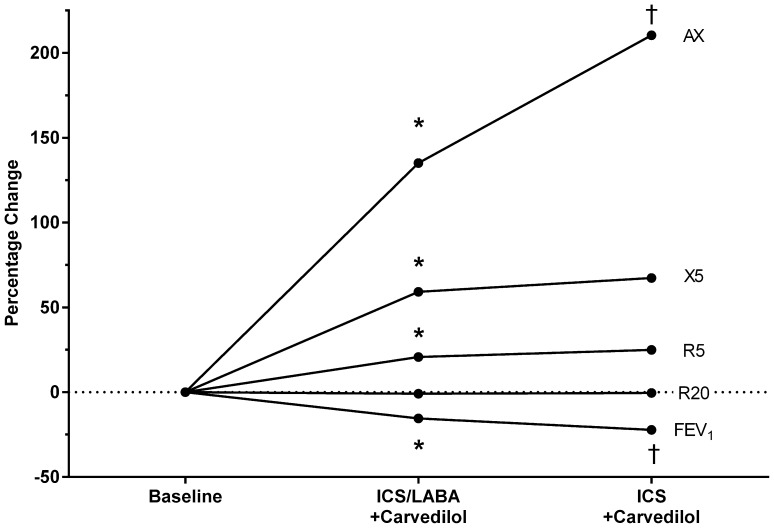
Comparison of percentage change from baseline (on ICS/LABA without beta-blocker) in reactance (i.e. compliance: AX, X5) and resistance (R5, R20), and forced expiratory volume in 1 s (FEV_1_). Asterisk denotes *P* < 0.05 versus baseline, cross denotes *P* < 0.05 versus ICS/LABA

Patients had a mean age 65 years, mean smoking pack year history of 53, mean FEV_1_ 52% predicted, and mean 7% reversibility of FEV_1_ to salbutamol 400 µg. Percentage change from baseline in IOS (R5, R20, X5, AX) and FEV_1_ are depicted in Fig. 1. In terms of airway resistance, R5 increased significantly from the presence of carvedilol, while no further increase in R5 occurred when the LABA was withdrawn. In contrast, there was no significant change in R20 at any point.

In terms of reactance, there was a significant increase (i.e. reduced compliance) in both X5 and AX in the presence of carvedilol, however only AX increased significantly more after LABA withdrawal. FEV_1_ decreased significantly in the presence of carvedilol and decreased significantly further after LABA withdrawal, while FVC was only reduced after LABA withdrawal. SRM’s (Table 1) for reactance were higher than those for resistance, with AX being greater than X5.

**Table 1:** Mean (95% CI) differences as change from baseline (on ICS/LABA without beta-blocker): in forced expiratory volume in 1 s (FEV1), and impulse oscillometry values of resistance (R) at 5 Hz (R5) and 20 Hz (R20) and reactance (X) at 5 Hz (X5) and area under the curve (AX), with standardised response means (SRM)

	Carvedilol + ICS/LABA		
	Mean difference (95% CI)	*P*	SRM
FEV1 (L)	− 0.21 (− 0.27, − 0.15)	< 0.001	2.21
FVC (L)	− 0.22 (− 0.44, − 0.01)	0.046	0.65
R5 (kPa/L/s)	0.10 (0.01, 0.19)	0.03	0.72
R20 (kPa/L/s)	− 0.01 (− 0.05, 0.03)	0.55	0.18
X5 (kPa/L/s)	− 0.10 (− 0.16, − 0.05)	< 0.001	1.19
AX (kPa/L/s Hz)	1.46 (0.63, 2.29)	< 0.001	1.11

	Carvedilol + ICS		
	Mean difference (95% CI)	*P*	SRM
FEV1 (L)	− 0.30 (− 0.39, − 0.20)	< 0.001	2.08
FVC (L)	− 0.60 (− 0.85, − 0.35)	< 0.001	1.52
R5 (kPa/L/s)	0.11 (0.01, 0.21)	0.03	0.72
R20 (kPa/L/s)	− 0.02 (− 0.07, 0.03)	0.50	0.20
X5 (kPa/L/s)	− 0.10 (− 0.17, − 0.03)	0.01	0.90
AX (kPa/L/s Hz)	1.70 (1.08, 2.32)	< 0.001	1.74

The higher the SRM the better the signal to noise ratio for a given variable. Mean baselines values were as follows: FEV1 1.43 L, FVC 3.55 L, R5 0.60 kPa/L/s, R20 0.39 kPa/L/s, X5 − 0.27 kPa/L/s, AX 2.69 kPa/L/s Hz

## Discussion

In patients receiving ICS/LABA, carvedilol challenge produced no significant change in R20, in turn suggesting that larger airways are not involved in β2-mediated bronchoconstriction in COPD. Previous data have also shown that R20 is unrelated to airflow obstruction or lung hyperinflation in COPD [3, 8]. It is known that LABA reduces lung hyperinflation [9] as measured by RV, and that in turn reactance is related to RV—in other words hyperinflation and compliance appear to go hand in hand [10]. This is supported by using FVC as a surrogate for air trapping in COPD, where our data showed a 17% decrease in FVC comparing ICS alone to ICS/LABA in the presence of carvedilol. It is worth pointing out that the presence of emphysema would be expected to increase compliance due to loss of elastic lung recoil [11]. However, emphysema also results in reduced airway support due to loss of alveolar attachments, manifesting as small airway closure. Hence the net result of reduced compliance presumably reflects the relative dominance of small airways closure in response to beta-2 antagonism and LABA withdrawal. The finding of increased small airway resistance was evident as heterogeneity of resistance between 5 and 20 Hz (R5-20) is also in keeping with alerted small airways geometry.

In terms of bronchodilator response in COPD, tiotropium and tulobuterol have been shown to produce significantly additive effects on X5, AX, R5 but not R20 [8]. Accordingly in terms of bronchoconstriction in our patients, there was a significant increase in R5, but not R20, and an increase in AX, and X5. This was in keeping with the ECLIPSE cohort, where AX rather than R5 more closely followed COPD severity defined by FEV1, while R20 was unchanged [2]. Our data were also similar to in TORCH [12], where ICS/LABA was significantly different to ICS alone on FEV_1_. This is perhaps unsurprising since our patients were selected a priori on the basis of a fall in FEV1 while taking ICS/LABA with carvedilol. In terms lung compliance, both AX and X5 increased significantly after carvedilol; however, only AX significantly increased after removal of LABA. AX represents lung compliance component of low-frequency-dependent reactance, and appears to be more sensitive than reactance measured at 5 Hz (X5), for identifying increasing changes in response to combined β_2_-receptor antagonism and agonism.

The limitations of our analysis are its small sample size, and lack of corroboration of reactance (AX, X5) with static lung volumes such as RV/TLC ratio. Moreover, we did not measure the within breath differences between inspiratory and expiratory reactance as a measure of dynamic hyperinflation, for the pragmatic reason that this is not performed in our routine clinical practice. We also acknowledge that we did not perform CT imaging to define the degree of emphysema nor did we evaluate ventilation heterogeneity using multiple breath nitrogen washout. Nonetheless, using FVC as a crude surrogate for air trapping, we did observe a significant reduction in response to carvedilol in conjunction with LABA withdrawal.

This analysis provides evidence that reduced lung compliance (as AX) measured by IOS is a sensitive outcome in response to bronchoconstriction in COPD. It would be relevant to know if exacerbation frequency and symptoms are more closely related to alterations in compliance than resistance, and in turn whether lung compliance might be a potential target for long acting bronchodilator therapy, for example comparing ICS/LABA/LAMA to ICS/LABA.
